# Clinical Characteristics of Anti-*N*-Methyl-d-Aspartate Receptor Encephalitis Overlapping with Demyelinating Diseases: A Review

**DOI:** 10.3389/fimmu.2022.857443

**Published:** 2022-06-28

**Authors:** Shujiang Zhang, Yuan Yang, Wenyu Liu, Zuoxiao Li, Jinmei Li, Dong Zhou

**Affiliations:** ^1^ Department of Neurology, West China Hospital of Sichuan University, Chengdu, China; ^2^ Department of Neurology, The Affiliated Hospital of Southwest Medical University, Luzhou, China

**Keywords:** aquaporin-4-antibody-positive neuromyelitis optica spectrum disorder, demyelinating diseases anti-N-methyl-D-aspartate receptor encephalitis, myelin oligodendrocyte glycoprotein antibody-associated disease, multiple sclerosis, overlapping syndromes

## Abstract

Anti-*N*-methyl-d-aspartate receptor encephalitis (NMDARe), a common autoimmune encephalitis, can be accompanied by demyelinating disorders, including multiple sclerosis (MS), neuromyelitis optica spectrum disorder (NMOSD), and myelin oligodendrocyte glycoprotein antibody-associated disease (MOGAD). To compare the clinical characteristics of patients with different overlapping syndromes, we searched the PubMed database and performed a systematic review. Of the 79 patients with overlapping syndromes, 15 had MS, 18 had aquaporin-4-antibody-positive NMOSD (AQP4-Ab-positive NMOSD), and 46 had MOGAD. Compared with classical NMDARe, overlapping syndromes showed atypical symptoms, such as limb weakness, sensory disturbance, and visual impairments in addition to the main symptoms of NMDARe and a lower ratio of ovarian teratoma. Patients with MOGAD overlap were the youngest, while patients with MS and AQP4-Ab-positive NMOSD overlap tended to be older than patients with classical NMDARe. A majority of patients with NMDARe who overlapped with MS or AQP4-Ab-positive NMOSD were female, but this was not the case for patients overlapped with MOGAD. When NMDARe and demyelinating diseases occurred sequentially, the interval was the longest in patients with NMDARe overlapped with MS. A favorable outcome was observed in patients overlapping with MOGAD, but no robust comparison can be drawn with the patients overlapping with AQP4-Ab-positive NMOSD and MS regarding the small number of available data. The long-term prognosis of overlapping syndromes needs further investigation.

## Introduction

Anti-*N*-methyl-d-aspartate receptor encephalitis (NMDARe) is a common autoimmune encephalitis with primary symptoms comprising cognitive dysfunction, psychiatric disorders, seizures, dyskinesia, decreased consciousness, speech disturbances, autonomic nervous dysfunctions, and central hypoventilation caused by cortical impairment (1). Multiple sclerosis (MS), aquaporin-4-antibody-positive neuromyelitis optica spectrum disorder (AQP4-Ab-positive NMOSD), and myelin oligodendrocyte glycoprotein antibody-associated disease (MOGAD) are distinct inflammatory demyelinating disorders of the central nervous system (2, 3). Recently, cases of NMDARe overlapping with demyelinating diseases have been reported; however, most were presented as case reports, cases series, or observational studies with small sample sizes. Several reviews have summarized the overlap of NMDARe with NMOSD or MOGAD (3–5), but it remains unknown whether there are differences in clinical features among the three overlapping syndromes. Therefore, we have reviewed NMDARe overlapped with MS, aquaporin-4-antibody-positive NMOSD (AQP4-Ab-positive NMOSD), and MOGAD, focusing on clinical differences and comparing the overlapping syndromes with classical NMDARe. The immune mechanisms thought to be involved in the overlapping syndromes are discussed.

## Literature Search Strategy

**Figure 1:**
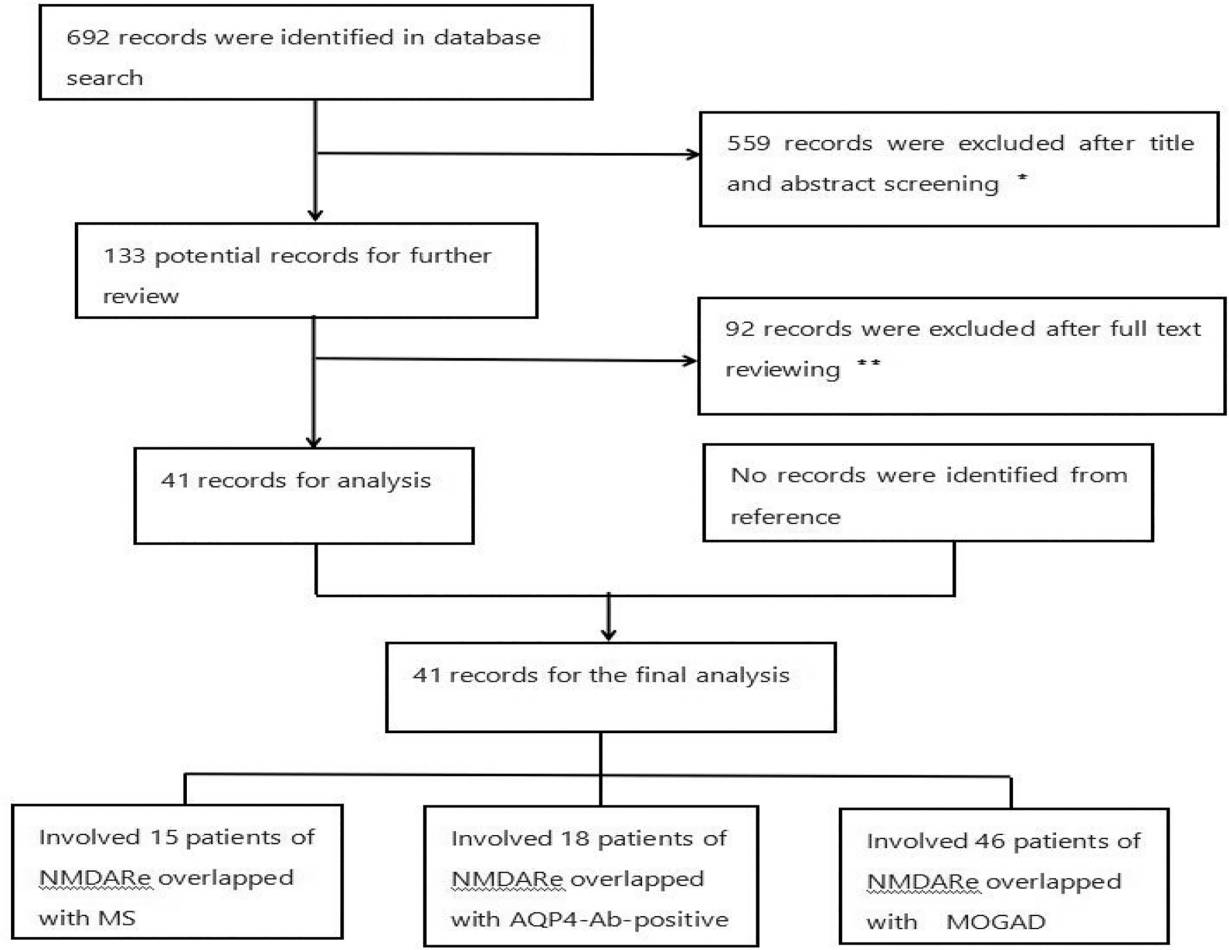
Flowchart of the study. *Reviews and articles describing patients with a single disease were excluded. **Articles describing patients without key information or aquaporin-4-antibody-negative NMOSD were excluded. There were 15, 18, and 46 NMDARe patients overlapped with MS, AQP4-Ab-positive NMOSD, and MOGAD, respectively.

The literature search strategy was performed using the Preferred Reporting Items for Systematic Reviews and Meta-Analyses (PRISMA) guidelines. PubMed was searched for the terms “NMDAR” and “MS,” “NMDAR” and “NMOSD,” “NMDAR” and “MOG,” “NMDAR” and “AQP4,” “NMDAR” and “demyelination,” “MS” and “demyelination,” “AQP4” and “NMOSD,” and “MOG” and “encephalitis” from inception to July 2021 to identify NMDARe cases overlapped with MS, AQP4-Ab-positive NMOSD, and MOGAD. Case reports and observational studies were included. The inclusion criteria were the following (1): NMDARe (2); clinical and/or MRI findings compatible with MS or AQP4-Ab-positive NMOSD, or MOGAD; and (3) seropositivity for MOG-IgG or AQP4-IgG and cerebrospinal fluid positivity for NMDA receptor antibodies, with positivity confirmed by cell-based assays. Patients with NMOSD diagnosis criteria but serum negative for AQP4-IgG and those who did not present clinical symptoms or radiological signs for MOGAD/NMOSD were excluded (**Figure 1**). We summarize the demographics, clinical presentations, laboratory data, treatments, and outcomes of patient with overlapping syndromes.

## Results

**Figure 2:**
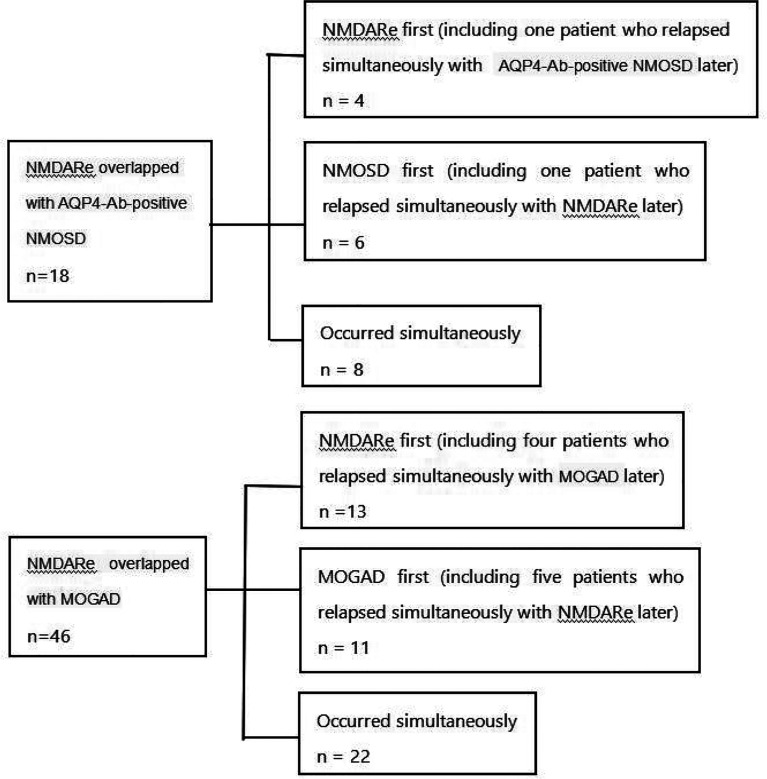
Details of the sequence of onset in patients with overlapping AQP4-Ab-positive NMOSD or MOGAD. NMDARe, anti-N-methyl-d-aspartate receptor encephalitis; AQP4-Ab-positive NMOSD, aquaporin-4-antibody-positive neuromyelitis optica spectrum disorder; MOGAD, myelin oligodendrocyte glycoprotein antibody-associated disease; NMOSD, neuromyelitis optica spectrum disorder.

Our search identified 79 patients with NMDARe and overlapping demyelinating disease from 30 individual case reports, 2 case series, and 9 observational studies (2–4, 6–43). Clinical data are summarized in **Tables 1**, **2** and **Figures 2** **–** **4**. The majority (62%) of patients was female, the median age was 26.5 years (27.5 ± 14.89), and the age of onset ranged from 4 to 63 years.

**Table 1:** Demographics, clinical characteristics, laboratory data, and treatments in the first NMDARe episode of three groups.

Characteristic	NMDARe-MS(n = 15)	NMDARe-AQP4-Ab-positive NMOSD(n = 18)	NMDARe-MOGAD(n = 46)	p
Median age^a^, years (range)	32 (17–52)	31 (13–62)	21 (4–63)	0.053
Male	3/15	1/18	26/46	<0.001
Tumor	0	2/18	0	0.121
NMDARe first	4/15	4/18	13/46	0.939
Required intensive care and ventilatory support	4/15	1/18	4/46	0.083
Abnormal MRI	13/15	18/18	44/46	0.267
Infratentorial or spinal cord abnormalities	5/15	6/18	17/46	1.000
multifocal or extensive T2-FLAIR abnormalities or white matter involvement	11/15	14/18	35/46	1.000
First-line treatment	12/15	17/18	43/46	0.120
Second-line treatment	7/15	10/18	17/46	0.421
Developed NMDARe and a demyelinating episode simultaneously^d^	–	10/18	31/46	0.399
Interval^a^ median (range, months)	41.5 (16–180)	33.5 (10–84)	12 (1–252)	0.037
mRS max, NMDARe median (range)^b^	–	5 (3–5)	4 (2–5)	0.051
Follow-up time, months^c^ (median)	18	12	12	0.797
mRS ≤ 2^e^	5/6	9/15	39/39	<0.001
Relapses NMDARe Demyelination NMDARe + demyelination (separately and/or simultaneously)	5/15 1 4 0	5/18 1 3 1	15/46 4 7 4	0.946

AQP4-Ab-positive NMOSD, aquaporin-4-antibody-positive neuromyelitis optica spectrum disorder; MOGAD, myelin oligodendrocyte glycoprotein antibody-associated disease; mRS, modified Rankin scale; MS, multiple sclerosis; NMDARe, anti-N-methyl-d-aspartate receptor encephalitis

When a demyelination episode occurred prior to NMDARe, the age at NMDARe onset is presented. We tested unlabeled data with the Fisher–Freeman–Halton extension of the Fisher exact test.

a Age and time interval of onset were compared by the Kruskal–Wallis test; when patients had multiple episodes of NMDARe or demyelinating episodes, only the longest interval between the two events was calculated.

b mRS was compared by the independent sample t-test. Only patients with recorded mRS data were considered.

c Follow-up time was compared by one-way analysis of variance.

d Patients developed NMDARe and a demyelinating episode simultaneously.

e Due to the inconsistency in follow-up time in each study or the lack of specific follow-up time in some of the studies, only patients with a clear mRS score or complete remission of symptoms (inferred mRS=0) were analyzed.

**Table 2:** Summary of clinical symptoms of the three groups.

Symptoms	NMDARe-MS	NMDARe-AQP4-Ab-positive NMOSD	NMDARe-MOGAD*	p
**Prodromal symptoms**	5/15	5/18	22/45	0.289
Signs of infection	4/15	5/18	22/45	0.170
Fatigue	2/15	0	0	**0.035**
**Cognitive dysfunction**	8/15	10/18	19/45	0.577
Memory loss	4/15	8/18	11/45	0.322
Disorientation	3/15	None	2/45	0.059
Execution	2/15	None	None	**0.035**
Acalculia	2/15	None	None	**0.035**
Thought disorganization	1/15	None	None	0.192
Attention deficit	None	1/18	None	0.418
Unclassified	2/15	3/18	7/45	–
**Psychiatric disorder**	11/15	16/18	31/45	0.270
Abnormal behavior	7/15	11/18	15/45	0.122
Agitation	1/15	2/18	None	0.072
Mood disorders	3/15	6/18	12/45	0.688
Psychosis	5/15	4/18	9/45	0.574
Hallucinations	3/15	None	4/45	0.118
Delusions	4/15	None	2/45	**0.016**
Unclassified	None	None	4/45	–
**Seizures**	8/15	5/18	25/45	0.126
Focal seizures	1/15	–	1/45	–
Tonic-clonic seizures	3/15	–	–	–
Status epilepticus	2/15	None	None	**0.035**
Unclassified	5/15	5/18	24/45	–
**Dyskinesia**	8/15	5/18	16/45	0.330
Gait disturbance/ataxia	1/15	2/18	4/45	1.000
dystonia	None	1/18	None	0.407
Tremor	1/15	1/18	None	0.176
Involuntary movement	4/15	4/18	6/45	0.397
Unclassified	4/15	3/18	7/45	–
**Decreased consciousness**	10/15	8/18	17/45	0.160
Confusion	6/15	1/18	1/45	**0.001**
Somnolence	None	3/18	6/45	0.299
Coma	2/15	2/18	3/45	0.639
Unclassified	3/15	3/18	7/45	–
**Speech disturbances**	3/15	9/18	14/45	0.208
Aphasia	None	1/18	2/45	1.000
Dysfluency	3/15	8/18	12/45	0.287
**Autonomic nervous dysfunctions**	2/15	4/18	2/45	0.065
**Central hypoventilation**	3/15	3/18	7/45	0.916
**Sleep dysfunction**	1/15	4/18	14/45	0.145
**Dysarthria**	1/15	1/18	3/45	1.000

For patients who had multiple episodes, only the symptoms at the time of first NMDARe diagnosis were included in the analysis. AQP4-Ab-positive NMOSD, aquaporin-4-antibody-positive neuromyelitis optica spectrum disorder; MOGAD, myelin oligodendrocyte glycoprotein antibody-associated disease; MS, multiple sclerosis; NMDARe, anti-N-methyl-d-aspartate receptor encephalitis

*Symptom details were only included for 45 patients because the report of one patient only briefly mentioned NMDARe as part of their medical history (involved in reference 38), and no relevant clinical symptoms of NMDARe were described. The bold values provided in Table means differences was statistically significant.

**Figure 3:**
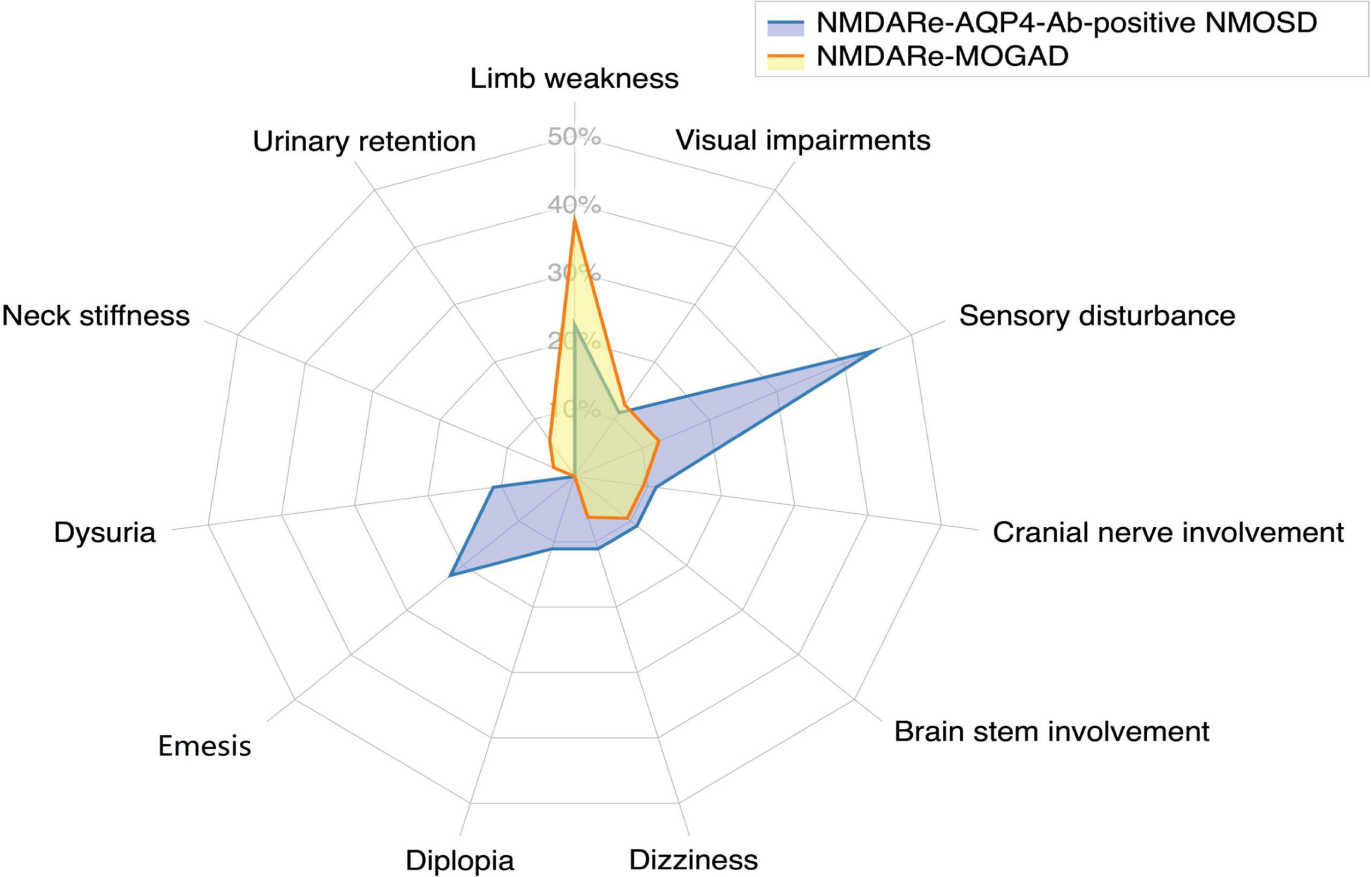
Frequencies of atypical symptoms. Sensory disturbance was the most common atypical symptom in patients overlapping with AQP4-Ab-positive NMOSD; limb weakness was the most common atypical symptom in patients overlapping with MOGAD.

**Figure 4:**
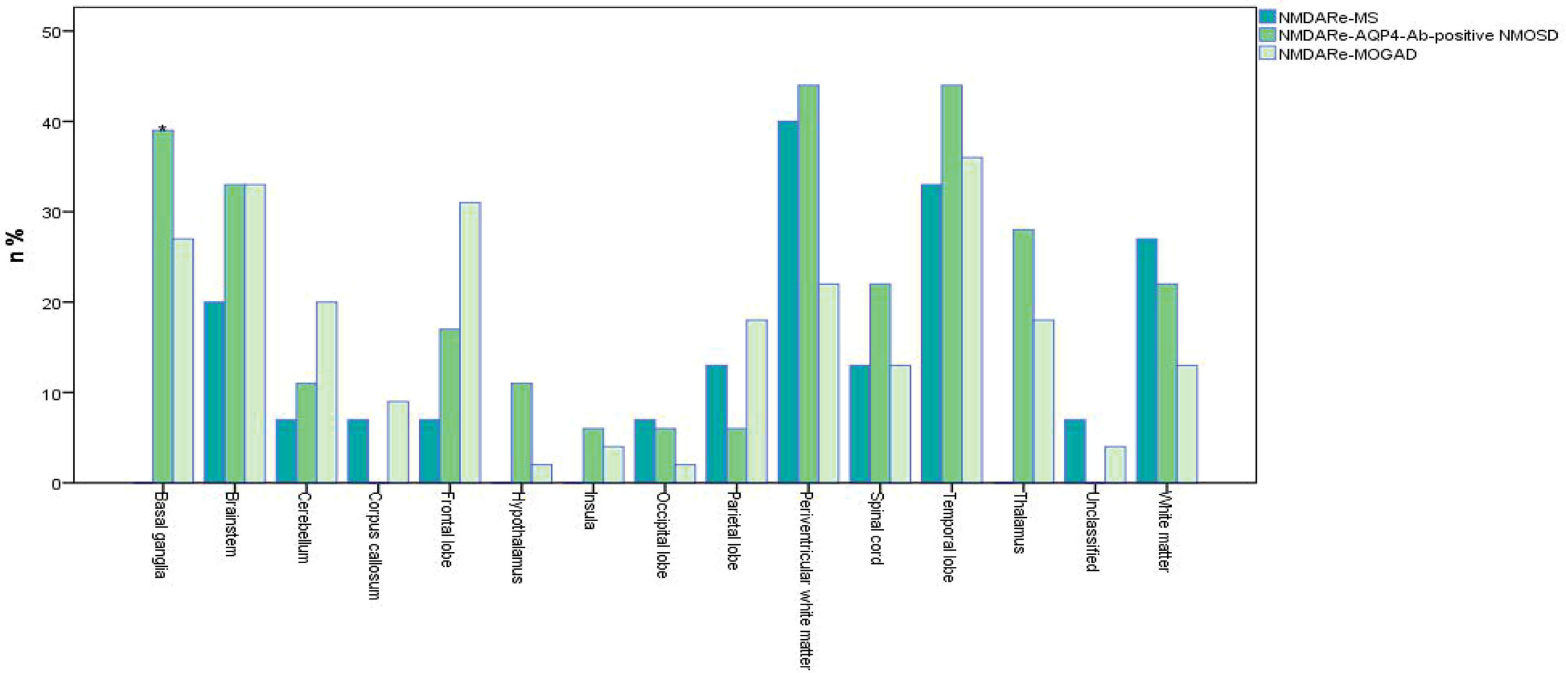
Frequencies of MRI lesions in overlapping syndromes. *No basal ganglia involvement in patients overlapped with MS, *p* = 0.016. Unclassified meant no specific details of MRI abnormalities in original references. White matter refers to all parts of the brain except the paraventricular white matter. Multifocal, infratentorial, and spinal cord involvement all were common in the three overlapping syndromes. Periventricular white matter was the most common lesion site in NMDARe overlapping with MS; periventricular white matter and the temporal lobe were the most common lesion sites in NMDARe overlapping with AQP4-Ab-positive NMOSD; the temporal lobe was the most common lesion site in NMDARe overlapping with MOGAD.

### NMDARe Overlapping With MS

In a total of 15 patients with NMDARe overlapping with MS, relapse–remitting MS was reported in 11 patients and an unknown subtype in the other four patients. Among them, 11 patients had MS onset prior to NMDARe, and the median interval between NMDARe onset and MS was 41.5 months. Three patients were male, the median age was 32 years, and the age of onset ranged from 17 to 52 years. None of the patients were diagnosed with a tumor.

Five patients had prodromal symptoms prior to onset of NMDARe (5/15), including headache and/or fever in four patients and fatigue in two patients. Patients overlapped with MS presented abnormal behavior (7/15), psychosis (5/15), delusions (4/15), hallucinations (3/15), mood disorders (3/15), and agitation (1/15). Decreased consciousness was reported in 10 patients, and two of these progressed to coma. Cognitive dysfunction was reported in eight patients, and dyskinesia was reported in eight patients. Memory loss and involuntary movement were common symptoms in cognitive dysfunction and dyskinesia. Eight patients had seizures, and two patients had status epilepticus. Speech dysfluency instead of aphasia was observed in three patients. In addition, three patients had central hypoventilation, two patients had autonomic nervous dysfunctions, one patient had sleep dysfunction, and one patient had dysarthria.

Positive oligoclonal bands in cerebrospinal fluid (CSF) was reported in seven patients. Other autoimmune antibodies were detected, including serum anti-nuclear antibodies (ANA) in two patients, thyroglobulin antibody (TGA) in one patient, and thyroid peroxidase (TPO) in another patient. One patient had ANA, extractable nuclear antigen (ENA), and anti-ribonucleoprotein (RNP). Thirteen of 15 patients showed abnormal MRI results (**Figure 4**), and all of them had new lesions on MRI at the onset of NMDARe. Typical demyelination lesions were found on MRI at NMDARe diagnosis in five patients. The most common lesion site was within the periventricular white matter, and the second most common was in the temporal lobe. Eleven patients presented multifocal or extensive T2/fluid-attenuated inversion-recovery (FLAIR) abnormalities, and five patients presented infratentorial and/or spinal cord abnormalities. Eleven patients were diagnosed with MS before NMDARe, seven patients received disease-modifying treatment, and three of them received a variety of disease-modifying drugs. Three patients were treated with dimethyl fumarate, two patients were treated with interferon beta-1a, two patients were treated with natalizumab, and another five patients received interferon beta-1b, glatiramer acetate, fingolimod, teriflunomide, and daclizumab, respectively. Six of the seven patients received the aforementioned disease-modifying therapies in the relapsing course of MS. Twelve of the patients received either steroids (st), intravenous methylprednisolone (IVMP), intravenous immunoglobulins (IVIG), plasma exchange (PE), or a combination thereof. Seven patients received either rituximab (RTX), azathioprine (AZA), mycophenolate mofetil (MMF), cyclophosphamide (CTX), bortezomib, natalizumab, or mitoxantrone, or a combination thereof at the onset of NMDARe (**Supplementary Table S1**).

Four patients required intensive care and ventilatory support. Five patients were re-examined with MRI, three patients showed improvement of lesions, one patient showed complete remission of symptoms, whereas one patient showed progression of lesions and died. Details regarding response to immunotherapy were reported in just six patients; of these, five patients showed modified Rankin scale (mRS) ≤2 at the last follow-up (median, 18 months). Cognitive dysfunction in three patients, psychiatric disorder in one patient, and decreased consciousness in another patient were reported at the last follow-up. Five patients showed relapses during the follow-up period, one had an NMDARe relapse, and the other four had MS relapses.

### NMDARe Overlapped With AQP4-Ab-Positive NMOSD

A total of 18 patients with NMDARe were overlapped with AQP4-Ab-positive NMOSD. Among them, 10 patients developed NMDARe and NMOSD separately, and eight developed NMDARe and NMOSD simultaneously (**Figure 2**). The median interval between NMDARe onset and AQP4-Ab-positive NMOSD was 33.5 months. Only one patient was male. The median age was 31 years, and the age of onset ranged from 13 to 62 years. Two patients had ovarian teratoma.

Five patients had headache and/or fever prior to onset of NMDARe (28%). Patients overlapped with AQP4-Ab-positive NMOSD presented abnormal behavior (11/18), mood disorders (6/18), psychosis (4/18), and agitation (2/18). Cognitive dysfunction was reported in 10 patients, memory loss being the most frequent (8/18). Speech disturbances, decreased consciousness, and dyskinesia were reported in nine, eight, and five patients, respectively. Five patients had seizures during the disease course, but none had status epilepticus. In addition, four patients had autonomic nervous dysfunctions, four had sleep dysfunction, three had central hypoventilation, and one had dysarthria. When patients developed NMDARe and AQP4-Ab-positive NMOSD episodes simultaneously, atypical symptoms, such as sensory disturbances, limb weakness, and emesis, were reported (details are presented in **Figure 3**).

Positive oligoclonal bands in CSF was reported in four patients. Other autoimmune antibodies detected included serum ANA in one patient and anti-Sjögren syndrome A (anti-SSA) in another patient. MRI showed new lesions in all patients at the onset of NMDARe, except in one patient whose MRI lesion was left over from a previous demyelinating event. The periventricular white matter and temporal lobe were the most common lesion sites, followed by the basal ganglia. Fourteen patients presented multifocal or extensive T2/FLAIR abnormalities, and six patients presented infratentorial and/or spinal cord abnormalities.

Seventeen patients received either st, IVMP, IVIG, or PE, or a combination thereof. Ten patients received RTX, AZA, MMF, and CTX, or a combination thereof at the onset of NMDARe (**Supplementary Table S1**). A single patient required intensive care and ventilatory support. After treatment, lesions on MRI had subsided in five patients. One patient showed progression of lesions and subsequently died. Nine of 15 patients showed mRS ≤2 at the last follow-up (median, 12 months). No remaining disability was reported in 14 patients, and one patient had died as of the last follow-up. Five patients showed relapses during the follow-up period, one patient had an NMDARe relapse, three patients had NMOSD relapses, and one patient had repeated relapses of NMDARe and/or NMOSD.

### NMDARe Overlapped With MOGAD

A total of 46 patients with NMDARe were overlapped with MOGAD. Among them, 24 patients developed NMDARe and MOGAD separately and 22 patients developed NMDARe and NMOSD simultaneously (**Figure 2**). The median interval between NMDARe onset and MOGAD was 12 months. Twenty-six patients were male. The median age was 21 years, the age of onset ranged from 4 to 63 years. None of the patients was diagnosed with a tumor.

Symptom details were only included for 45 patients because the report of one patient only briefly mentioned NMDARe as part of their medical history, and no relevant clinical symptoms of NMDARe were described. Twenty-two patients had headache and/or fever prior to onset of NMDARe (49%). Patients overlapped with MOGAD presented abnormal behavior (15/45), mood disorders (12/45), psychosis (9/45), hallucinations (4/45), agitation (2/45), and delusions (2/45). Seizures were reported in 25 patients, none of whom had status epilepticus. Cognitive dysfunction, decreased consciousness, and dyskinesia were reported in 19, 17, and 16 patients, respectively. Fourteen patients had speech disturbances. In addition, 14 patients had sleep dysfunction, seven patients had central hypoventilation, three patients had dysarthria, and two patients had autonomic nervous dysfunctions. When patients developed NMDARe and MOGAD episodes simultaneously, atypical symptoms, such as limb weakness, sensory disturbance, and visual impairment were reported (details are presented in **Figure 3**).

Positive oligoclonal bands in CSF was reported in 10 patients. Other autoimmune antibodies were detected, including serum ANA in three patients, TPO in one patient, TGA and TPO in four patients, voltage-gated potassium channel complex (VGKC) in one patient, contactin-associated protein-like 2 (CASPR2) in one patient, ANA and anti-SSA/anti-Sjögren syndrome B (anti-SSB) in one patient, and anti-SSA in one patient. Forty-four patients showed MRI abnormalities (**Figure 4**), all of which were new lesions at the onset of NMDARe. The temporal lobe was the most common lesion site, followed by the brainstem. Thirty-five patients presented multifocal or extensive T2/FLAIR abnormalities, and 17 patients presented infratentorial or spinal cord abnormalities.

Forty-three patients received either st, IVMP, IVIG, or PE, or a combination thereof. Seventeen patients received RTX, MMF, AZA, or natalizumab, or a combination thereof at the onset of NMDARe (**Supplementary Table S1**). Four patients required intensive care and ventilatory support. After treatment, 14 patients were re-examined with MRI. Six showed improvement of lesions, and the other eight showed completely subsided lesions. Details regarding response to immunotherapy were reported for 39 patients only; all had mRS ≤2 at the last follow-up (median, 12 months). Psychiatric disorder was observed in three patients, cognitive dysfunction in two patients, seizures in two patients, and hemiparesis in one patient at the last follow-up. Relapses were observed in 15 patients (four patients had NMDARe relapse, seven patients had MOGAD relapse, four patients had repeated relapses of NMDARe and/or MOGAD.

### Differences Between Overlapping Syndromes

The three overlapping syndromes evaluated in this study showed different clinical features. The youngest age at onset was observed in patients with NMDARe overlapping with MOGAD. The majority of patients with NMDARe overlapped with MS, and AQP4-Ab-positive NMOSD were female, but this was not the case for patients overlapped with MOGAD (*p* < 0.001). Except for two patients overlapped with AQP4-Ab NMOSD with ovarian teratoma, tumor occurrence was not observed in any NMDARe patients overlapping with demyelinating diseases. The interval between NMDARe and demyelinating diseases was the longest for NMDARe overlapping with MS among the three groups (*p* = 0.037). Symptoms of fatigue, execution, acalculia, status epilepticus, and confusion were more common in patients overlapped with MS than in the other two groups. Patients overlapped with AQP4-Ab-positive NMOSD had fewer delusion symptoms than the other two groups. There was no significant difference in atypical symptom frequency between overlap with AQP4-Ab-positive NMOSD or MOGAD.

There was no basal ganglia involvement in patients overlapped with MS, while basal ganglia involvement was obvious in patients overlapped with AQP4-Ab-positive NMOSD and MOGAD (*p* = 0.016).

Use of intensive care and respiratory support was not different among the three groups, and no differences in treatment strategies were reported. Patients overlapping with MOGAD responded favorable to immunotherapy, since 39 patients who had details regarding response to immunotherapy all showed mRS ≤2. However, no robust comparison can be drawn with the patients overlapping with AQP4-Ab-positive NMOSD and MS regarding the small number of available data. Cognitive dysfunction and psychiatric disorder were reported as main sequelae in patients overlapped with MS and MOGAD. Sequelae details were not reported in most patients overlapped with AQP4-Ab-positive NMOSD. No differences in relapse rate were observed among the three overlapping syndromes.

## Discussion

Reports of NMDARe overlapping with demyelinating diseases are increasing; therefore, overlapping syndromes may be a new entity that is different from NMDARe or demyelinating disease alone. Compared with classical NMDARe, overlapping syndromes have their own clinical characteristics. In a large cohort study of 577 patients with classical NMDARe, whose median age at onset was 21 years (44), we noted that patients with MS or AQP4-positive NMOSD overlap tended to be slightly older than those with classical NMDARe. In terms of the order in which NMDARe and demyelinating diseases appear, it is not clear whether NMDARe triggers demyelinating diseases, or *vice versa*. Among the three overlapping syndromes, the interval between MS and NMDARe onset was the longest. The female prevalence of MS and AQP4-positive NMOSD overlap was similar to that of classical NMDARe, but this was not the case for MOGAD. Ovarian teratoma occurrence in overlapping syndromes was much lower than the 58% teratoma occurrence in women of childbearing age with classical NMDARe (44). The two patients with ovarian teratoma who were included in our review had no differences in clinical symptoms from those without such tumors. Both patients underwent surgical resection and first-line immunotherapy and had relatively good prognoses, which indicates the value of combined surgery and immune therapy (2, 21). This indicates that the active autoimmune pathway may not be associated with a paraneoplastic pathway triggered by a tumor. Previous studies also suggested a low teratoma occurrence rate in NMDARe and demyelinating disease overlap patients with glial or neuronal surface antibodies (2, 45).

There was no significant difference in the main symptoms among the three groups of patients with NMDARe. Sensory disturbance was the most common atypical symptom of patients overlapping with AQP4-positive NMOSD, and limb weakness was the most common atypical symptom of patients overlapping with MOGAD. However, there was no obvious difference in terms of all atypical symptoms in patients overlapping with AQP4-Ab-positive NMOSD or MOGAD. In addition to cortical dysfunction, overlapping syndrome patients frequently present limb weakness, sensory disturbances and visual impairment, which may be attributed to subcortical lesions caused by demyelinating disease. However, the severity index of clinical symptoms and signs in patients with overlapping syndromes was relatively mild compared with patients with classical NMDARe, who frequently require intensive care in acute phases (1).

Abnormal MRI results showed greater multifocal subcortical lesion involvement in overlapping syndrome patients compared with classical NMDARe. The white matter (subcortical, periventricular, and deep), infratentorial, and the spinal cord were frequently involved in overlapping syndromes. In general, overlapping syndrome patients display conspicuous MRI changes. In contrast, only a third of patients with classical NMDARe display abnormal MRI findings, frequently involving lesions of the cortex, leptomeninges, and frontal, temporal, and limbic lobes (43, 46, 47). The difference in these MRI findings may be associated with differences in antigens and cell types between overlapping syndromes and classical NMDARe. It is worth noting that many overlapping syndrome patients showed white matter involvement or presented classical demyelinating changes on MRI at NMDARe diagnosis (4, 9, 13, 14, 31). This indicates that MRI abnormalities in white matter may be an early sign of overlapping syndrome, prior to positivity for AQP4 or MOG antibodies.

The evolution of NMDARe overlapped with demyelinating diseases is complex. At present, the specific mechanism of overlapping syndrome has not been fully elucidated. Our review found that some patients had signs of infection (headache and/or fever) prior to the onset of overlapping syndromes, especially in patients overlapped with MOGAD. This phenomenon is consistent with previous studies, which indicate that infection may be implicated in the dual positivity of anti-NMDAR/MOG antibodies (5). However, it is worth noting that many patients with overlapping syndromes were accompanied by other autoimmune antibodies, indicating a spread of autoimmune pathway activation in overlapping syndromes. Meanwhile, the use of immunomodulatory agents may trigger the generation of NMDA receptor or AQP4 or MOG antibodies or lead to immune dysregulation in overlapping syndromes (19). The same NMDAR and MOG autoantigens on the surface of oligodendrocytes may be the reason that they are stimulated together or sequentially (5, 48, 49). Further studies are needed to determine the associations of dual antibodies and specific immune mechanisms of overlapping syndromes.

The complexity of overlapping syndrome can make clinical diagnosis difficult. When a demyelinating episode occurs prior to NMDARe, symptom recurrence may be misdiagnosed as a demyelinating disease relapse because of its high recurrence rate. However, when a patient with a demyelinating disease presents prominent psychiatric symptoms, impairment of consciousness, orofacial dyskinesias, and/or autonomic dysfunction, testing for NMDAR antibodies should be considered. Similarly, when a patient with NMDARe presents with atypical symptoms and/or MRI lesions in the multifocal subcortical or infratentorial or white matter, the possibility of an overlapping demyelination episode should be considered. A comprehensive workup and searching for OB, AQP4, and MOG antibodies is highly recommended (2). However, when patients develop NMDARe and demyelinating disease simultaneously, the diagnosis is difficult. Some patients are positive for anti-AQP4 or anti-MOG antibodies without atypical symptoms and/or abnormal MRI findings (2, 28, 45), indicating that the presence of autoantibodies is not always a trigger of imminent demyelinating symptoms. Long-term observation is needed to determine whether these patients will develop demyelinating symptoms.

There was no difference between the use of first- and second-line treatments among the three overlapping syndrome groups. A previous study suggested that MOG antibodies may reflect an underlying pathogenic mechanism or even have a beneficial effect in demyelinating disorders and usually respond to immunotherapy (50, 51). This may also be the case in overlapping syndromes; patients overlapping with MOGAD responded favorable to immunotherapy. According to the available data, cognitive dysfunction and psychiatric disorders are the main sequelae of NMDARe overlapping demyelinating syndromes, and these symptoms seemed more likely to be caused by NMDARe. However, because there was a high rate of missing follow-up data for patients with AQP4-Ab-positive NMOSD and MOGAD, the differences in sequelae and long-term prognoses among the three groups remain unknown. Long-term follow-up investigation is thus needed. Second-line treatments are recommended in patients with overlapping demyelinating disease, which are considered to improve prognosis and lower the risk of relapse (26, 52, 53). However, there are no guidelines for the treatment of overlapping syndromes, and it is also unclear whether treatment should differ between different overlapping syndromes.

Although the initial immunotherapy treatments are similar for NMDARe and demyelinating disease, the subsequent treatments and prognoses are different. Most patients with NMDARe responded to first-line treatments, which resulted in mild residual deficits (1, 44). However, relapse of demyelinating diseases is common and frequently results in serious sequelae in long-term follow-up (2). Prompt diagnosis and treatment of overlapping demyelinating diseases should be emphasized.

A limitation of our study is that the data were collected retrospectively from different countries and regions. An assessment of long-term prognosis and relapse rates was not performed because of the small sample size, missing follow-up data, and short follow-up duration. Thus, differences in prognosis between the three groups remain unknown, as do treatment recommendations.

## Conclusions

NMDARe may occur simultaneously or sequentially with different demyelinating diseases. Compared with the age of patients with classical NMDARe, patients with overlapping MS or AQP4-positive NMOSD tended to be slightly older. They also had lower incidence of ovarian teratoma. Overlapping syndrome patients frequently presented atypical symptoms and/or MRI abnormalities when compared with classical NMDARe. MRI scans and detection of OB, AQP4, and MOG antibodies are helpful for identifying overlapping syndromes. Favorable outcome was observed in patients overlapping with MOGAD, but no robust comparison can be drawn with the patients overlapping with AQP4-Ab-positive NMOSD and MS regarding the small number of available data. Long-term follow-up investigation is needed because of the long interval between NMDARe and demyelinating diseases.

## Author Contributions

JL and DZ were responsible for designing the review. SZ performed data analysis and interpretation. YY helped with the analysis through constructive discussion. ZL helped with the literature review. All authors contributed to the article and approved the submitted version.

## Conflict of Interest

The authors declare that the research was conducted in the absence of any commercial or financial relationships that could be construed as a potential conflict of interest.

## Publisher’s Note

All claims expressed in this article are solely those of the authors and do not necessarily represent those of their affiliated organizations, or those of the publisher, the editors and the reviewers. Any product that may be evaluated in this article, or claim that may be made by its manufacturer, is not guaranteed or endorsed by the publisher.
