# Supplementary material for: Clinical Characteristics of Anti-N-Methyl-d-Aspartate Receptor Encephalitis Overlapping with Demyelinating Diseases: A Review
Source: Front Immunol. 2022 Jun 28;13:857443. doi: 10.3389/fimmu.2022.857443 (PMC9273846; doi:10.3389/fimmu.2022.857443)
Supplement: Supplementary file 1 [file Table_1.doc]

| Supplementary Table S1 Treatment details among the three groups | | | |
| --- | --- | --- | --- |
| Feature | NMDARe-MS  (n = 15) | NMDARe-AQP4-Ab-positiveNMOSD  (n = 18) | NMDARe-MOGAD  (n = 46) |
| First-line treatment  St only  IVIG only  IVMP only  St + IVIG  IVMP + PE  St + PE  St + IVMP  IVMP + IVIG  St + IVIG+ PE  IVMP + IVIG + PE  Untreated  Unknown | 12/15  3 /15  1/15  -  1/15  1/15  -  -  2 /15  -  4/15  1/15  2/15 | 17/18  2/18  -  3/18  3/18  -  -  -  5/18  4/18  -  0  1/18 | 43/46  5/46  1/46  8/46  4 /46  2/46  1/46  1/46  18/46  -  3/46  0  3/46 |
| Second-line treatment  RTX  CTX  MMF  AZA  A monoclonal Ab  CTX + RTX  AZA + RTX  AZA + MMF  RTX + bortezomib  MMF + CTX  RTX +MMF  MMF + natalizumab  CTX + AZA + natalizumab + mitoxantrone  Unused  Unknown | 7/15  3/15  -  -  -  1/15  -  -  1/15  1/15  -  -  -  1/15  6 /15  2/15 | 10/18  2/18  1/18  2/18  1/18  -  2/18  1/18  -  -  1/18  -  -  -  7/18  1/18 | 17/46  5/46  -  7/46  2/46  -  -  -  -  -  -  2/46  1/46 (2%)  -  26/46  3/46 |

All percentage values are rounded to an integer.

Ab = antibody; AQP4 = aquaporin-4; AQP4-Ab-positive NMOSD = AQP4-antibody-positive neuromyelitis optica spectrum disorder; AZA = azathioprine; CTX = cyclophosphamide; ENA = extractable nuclear antigen; IVIG = intravenous immunoglobulin; IVMP = intravenous methylprednisolone; MMF = mycophenolate mofetil; MOGAD = myelin oligodendrocyte glycoprotein antibody-associated disease; MS = multiple sclerosis; NMDARe = anti-N-methyl-d-aspartate receptor encephalitis; NMOSD = neuromyelitis optica spectrum disorder; PE = plasma exchange; RTX = rituximab; st = steroids.
